# Mechanism of Extracellular Vesicle Secretion Associated with TGF-β-Dependent Inflammatory Response in the Tumor Microenvironment

**DOI:** 10.3390/ijms232315335

**Published:** 2022-12-05

**Authors:** Klaudia Bonowicz, Klaudia Mikołajczyk, Inaz Faisal, Murtaz Qamar, Kerstin Steinbrink, Konrad Kleszczyński, Alina Grzanka, Maciej Gagat

**Affiliations:** 1 Department of Histology and Embryology, Collegium Medicum in Bydgoszcz, Nicolaus Copernicus University in Torun, 85-092 Bydgoszcz, Poland; 2 Department of Dermatology, University of Münster, Von-Esmarch-Str. 58, 48149 Münster, Germany

**Keywords:** inflammation, angiogenesis, transforming growth factor β, extracellular vesicles, tumor microenvironment

## Abstract

Extracellular vesicles (EVs) serve as central mediators in communication between tumor and non-tumor cells. These interactions are largely dependent on the function of the endothelial barrier and the set of receptors present on its surface, as endothelial cells (ECs) are a plenteous source of EVs. The molecular basis for EV secretion and action in the tumor microenvironment (TME) has not been fully elucidated to date. Emerging evidence suggests a prominent role of inflammatory pathways in promoting tumor progression and metastasis. Although transforming growth factor β (TGF-β) is a cytokine with strong immunomodulatory and protective activity in benign and early-stage cancer cells, it plays a pro-tumorigenic role in advanced cancer cells, which is known as the “TGF-β paradox”. Thus, the aim of this review is to describe the correlation between EV release, TGF-β-dependent inflammation, and dysregulation of downstream TGF-β signaling in the context of cancer development.

## 1. Introduction

The ability to communicate between cells is an absolute requisite for their proper functioning. Direct and indirect cell-to-cell signaling can be mediated through EVs. These submicron structures can act as shuttle vectors or signal transducers and orchestrate most pathophysiological processes, ranging from cell growth to tumor metastasis and invasion [1]. EVs are secreted by cells into the extracellular space and constitute a heterogeneous fraction enclosed by a lipid bilayer, which is the central part of the biological membrane surrounding the cytoplasmic part of the vesicle [2]. The fact that vesicles are secreted by both normal and cancer cells raises questions about their biological functions under the conditions of homeostasis and the course of pathogenesis [3]. To date, investigations have revealed that EV secretion is an integral part of the mechanism of discarding unwanted materials from cells. Thus, for an extended period, EVs have been equated with “plate dust” or “cell debris”, and they now constitute a breakthrough and a promising diagnostic marker of the 21st century [4].

In general, EVs vary in size, mode of release mechanism, content, function, and transported cargo, containing various proteins, lipids, and nucleic acids. The high functionality of EVs is owed to their small size, allowing them to move and mediate chemical signal transmission over short or long distances in vivo [5]. Their size ranges from 30 to 10,000 nm in diameter. Thus, the research literature classifies EVs into three main populations: exosomes, microvesicles (MVs), and apoptotic bodies; however, the mode of biogenesis, means of isolation, and cargo may prove more essential criteria in the future [6,7,8]. The effect of interactions mediated via EVs is dependent on both the donor and recipient cell type. These nano-sized subcellular structures can be associated with target cells in several ways, e.g., endocytosis, phagocytosis, or direct fusion with the cell membrane through receptor–ligand interactions. The mechanisms of EV secretion are of particular interest, owing to reports on the immunomodulatory activity of their content. According to the latest knowledge, transported functional biomolecules may modulate the immune system by affecting the pathways of inflammatory response [9,10].

The main inflammation trigger is the detection of infection or tissue damage by innate immune cells through the activation of pattern-recognition receptors (PRRs) [11]. The further stages of the inflammatory response aim to eliminate the factor damaging the tissue, which may be invasive pathogens, by lymphocyte recognition of self or foreign antigens. However, inflammation often begins for other reasons and it does not stop [12,13]. Chronic inflammation predisposes cancer development and supports all stages of tumorigenesis [14]. According to the latest scientific reports, cancerogenesis can also be promoted by EV secretion, as the presence of these particles is recognized as a marker of pathology development. In the TME, cells both release and receive EVs [15]. The cargo they carry and the proteins displayed on their surface shape the TME by promoting tumor cell growth and survival and enhancing the invasive/metastatic activity. The appreciation of the potential of EVs in cancer biology is reflected by the broad identification of the specific molecular charge, which involves proteins that regulate the transport and attachment of EVs to cells, proteins related to cell adhesion, proteins related to the migration process, and morphogenesis. Furthermore, EVs participate in angiogenesis, leading to tumor growth through vascularization, thereby increasing chronic inflammation [16,17]. The vascular endothelial growth factor (VEGF) and its receptor system, belonging to the tyrosine kinase family, are the primary factors regulating angiogenesis [18]. The tetraspanin family of proteins supports the VEGF packaging process into small EVs [19]. Consequently, EVs modulate the angiogenesis mechanism through the activation of vascular endothelial growth factor receptor-2 (VEGFR-2), which promotes the migration of ECs towards the cluster of mutant cells and leads to the formation of new blood vessels by providing nutrients to support cancerogenesis and growth of the tumor mass [20,21]. The angiogenesis process is also regulated by TGF-β expression, which is the crucial factor responsible for inducing the VEGF expression in Ecs. TGF-β signaling leads to opposing effects depending on activin receptor-like kinase-1 (ALK-1) and activin receptor-like kinase-5 (ALK-5) activity, which may lead to either inhibition or stimulation of proliferation, migration, and angiogenesis [22,23] in the same ambiguous way that TGF-β mediates most, if not all, tumor processes. On the one hand, TGF-β exerts a strong chemotactic effect on immune cells, directing them to the site of vasculogenesis [24]. On the other hand, this factor, along with other compounds, induces apoptosis of ECs and remodeling of the blood vessel network.

According to available data, only a few authors have highlighted the relationship between EV secretion and TGF-β-induced inflammatory changes. Thus, we are interested in summarizing the current understanding of the role of EVs and their cargo in the mediation of proinflammatory cellular signaling pathways. The aim of this review is to assess the functional impact of EVs on cancer cell biology and refer to the potential clinical uses of EVs in relation to their therapeutic roles.

## 2. The Immunomodulatory Role of TGF-β in the TME

TGF-β is a large family of cytokines named after its three prototypical isoforms, TGF-β1, TGF-β2, and TGF-β3. These variants are structurally and functionally similar but expressed in different tissues [25,26]. The most prevalent in solid tumors is TGF-β1, making it relevant to this review [27]. TGF-β is known for its high pleiotropy, i.e., the ability to exert various biological effects depending on the cell type and microenvironmental context [28,29,30]. TGF-β actions are highlighted in both tumor-suppressive and tumor-promoting mechanisms, which reveals its controversial and “treacherous” role in modulating cell fate [31]. In benign and early-stage cancer cells, the effect of TGF-β is dose-dependent, whereas a high concentration of cytokines leads to growth arrest. Accordingly, the level of TGF-β is maintained by a negative feedback mechanism. For this reason, in many sources, TGF-β is described as a protective cytokine. In turn, in advanced cancer cells, excessive TGF-β concentration results from a positive feedback loop and leads to tumor progression and metastasis [32]. This extreme dysregulation of TGF-β activity is well known in human cancer as the “TGF-β paradox” and is associated with a series of molecular events, the exact sequence of which remains to be fully elucidated.

The function of TGF-β in the tumor microenvironment is mostly the management of inflammatory and immune reactions that enhance tumor development [33]. Elevated production and accumulation of TGF-β1 lead to the loss of the characteristic phenotype of ECs in the endothelial to mesenchymal transition (EndMT) process, which contributes to their dysfunction [34,35]. ECs that undergo EndMT lose endothelial markers such as vascular endothelial cadherin (VE-cadherin) and gain markers of mesenchymal cells such as fibronectin (FN1) and N-cadherin (CDH2), resulting in actin cytoskeleton rearrangements and diminished intercellular adhesion. Consequently, they weaken their crucial selective barrier function and acquire increased proliferative and migratory capacity [36]. TGF-β-induced expression of mesenchymal markers is also observed in residing fibroblasts, epithelial cells, and pericytes [37]. Such conditions, combined with the induction of expression of adhesion molecules such as vascular cell adhesion molecule 1 (VCAM-1) and intercellular adhesion molecule 1 (ICAM-1) (also mediated by TGF-β), are conducive to immune cell infiltration into the tumor microenvironment [38]. Moreover, TGF-β can be excessively released by inflammatory cells and act in an autocrine/paracrine manner. TGF-β signaling mechanisms are observed in both adaptive and innate immunity [39]. TGF-β is known to suppress the activity of cytotoxic T-lymphocytes (CTLs) and promote the differentiation of regulatory T-cells (Tregs), which are a plentiful source of the TGF-β production and may further support CTL cell suppression while promoting Treg expansion. TGF-β is also a strong chemoattractant for neutrophil (N), natural killer (NK), monocyte (M), and macrophage (Mφ) cells. However, TGF-β has been shown to prevent NK-, N-, and Mφ-associated carcinoma cell death. The recruitment of immune cell populations and simultaneous suppression of their antitumor effector functions can result in a tumor microenvironment rich in immune-cell-derived growth factors and additional cytokines that further promote tumor progression and metastasis [40,41].

## 3. TGF-β Signal Transduction

**Figure 1:**
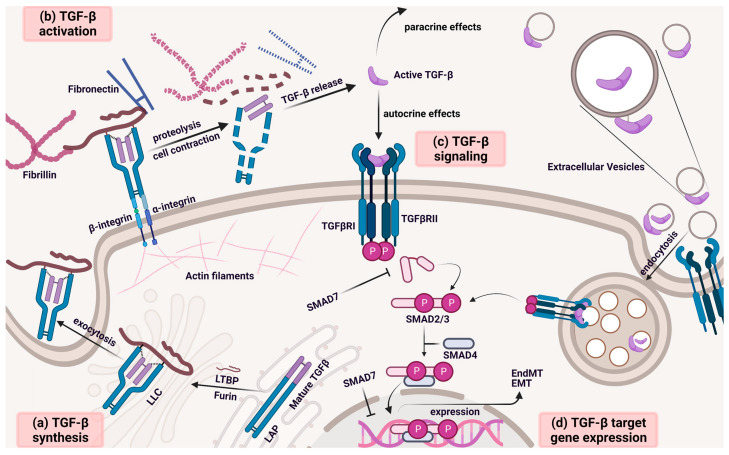
**TGF-β activation and canonical SMAD signaling.** The signaling of TGF-β family members is based on the activation of type I and type II dual-specificity serine/threonine kinase receptors at the surface of target cells. The type of activated receptor determines the final cellular response to active peptide ligand stimulation. (**a**) TGF-β is synthesized via ribosomes attached to the ER and then converted in the ER lumen to dimeric pro-TGF-β, the monomer of which consists of an LAP and a mature TGF-β fragment. Then, it crosslinks with the LTBP, forming an LLC. The dimer is cleaved by furin convertase in the Golgi complex, whereby it binds non-covalently to mature TGF-β and prevents TGF-β from binding to cell-surface receptors. Thereupon, LLC accumulates in secretory vesicles, which exocytose into the ECM. (**b**) The LLC is located in the ECM and crosslinks to fibrillin and fibronectin and binds to integrin receptors. The release and activation of mature TGF-β require a change in the LLC conformation triggered by a contraction force transmitted from integrin-associated actin filaments or proteolytic cleavage of fibrillin, fibronectin, LTBP, and LAP. (**c**) The active ligand of TGF-β is derived from ECM deposits or may be presented on the surface of EVs. Then, it binds to the type I and type II receptors for transforming growth factor β, which undergo transphosphorylation. The activated receptor complex initiates signal transduction by phosphorylation of receptor-regulated SMAD2 and SMAD3, which form heteromeric complexes with SMAD4. (**d**) The SMAD translocates to the nucleus, where it regulates the expression of target genes. Abbreviations: TGF-β, transforming growth factor β; SMAD, small mother against decapentaplegic; ER, endoplasmic reticulum; LAP, latency-associated peptide; LTBP, latent-transforming growth factor β-binding protein; LLC, large latent complex; ECM, extracellular matrix; EVs, extracellular vesicles; SMAD2, small mother against decapentaplegic family member 2; SMAD3, small mother against decapentaplegic family member 3; SMAD4, small mother against decapentaplegic family member 4.

The outcome of the cellular response to TGF-β depends on its signaling mechanisms, which are regulated extracellularly and intracellularly at multiple levels (Figure 1) [42]. TGF-β can react and be expressed in virtually every cell in the tumor environment [43]. Each of the three isoforms is coded by a separate gene on a different chromosome, but their sequences share an identity of as much as 71–79% in humans [44]. Secreted TGF-β is typically deposited in the extracellular matrix (ECM) as a part of a large latent complex (LLC) composed of TGF-β, latency-associated peptide (LAP), and a latent TGF-β-binding protein (LTBP) [45,46]. LAP propeptide binds to newly synthesized TGF-β via covalent and non-covalent linkage, and its function is to maintain the molecule in a conformation appropriate for dimerization and to keep it biologically inactive. LTBP connects with LAP through covalent bonding and targets and stabilizes LLC in fibrillin- and fibronectin-rich extracellular space [47,48]. The first biochemical event of TGF-β activation is the proteolytic degradation of fibrillin and fibronectin by elastase and bone morphogenetic protein 1 (BMP-1). The release of the mature ligand from its latent form requires proteolytic cleavage of LAP and LTBP, which can be stimulated by factors such as plasmin, cathepsin, matrix, and metalloproteinases. TGF-β release may also result from a distortion of the integrin-recognized LAP structure induced by actomyosin traction forces (Figure 1). The active form of TGF-β binds to a heterodimeric complex comprised of type I and type II TGFβ transmembrane serine–threonine receptors (TGF-βRI and TGF-βRII). TGF-βRIs are also referred to as activin receptor-like kinases (ALKs) [49]. TGF-β cellular responses are also regulated by TGF-βR-III (also known as betaglycan), which exhibits no enzymatic activity but is considered an important helper molecule that presents TGF-β to TGF-βR-II and facilitates its binding [50]. The following transphosphorylation of TGF-βRI induces phosphorylation of canonical downstream molecular mediators, receptor-activated small mothers against decapentaplegic (R-SMADs), such as SMAD2 and SMAD3. Low TGF-β concentrations in ECs can activate the SMAD1/5/8-based pathway. Activated R-SMADs form a complex with SMAD4 (Co-SMAD), enter the nucleus, and regulate transcription of TGF-β-responsive genes. The SMAD-independent pathways are also important for the response to TGF-β stimulation and include Src homology 2 domain-containing transforming protein 1 (ShcA), Ras homologous (Rho) protein family, Ras-related C3 botulinum toxin substrate (RAC), cell division control protein 42 homolog (CDC42), rat sarcoma virus (RAS) protein family, TNF-α receptor-associated factor 6 (TRAF6), transforming growth factor beta-activated kinase 1 (TAK1), phosphoinositide 3-kinase (PI3K), partitioning-defective protein 6 (PAR6), mitogen-activated protein kinase 1 (MAP3K1), death-associated protein 6 (DAXX), and protein phosphatase 2 (PP2A) [40].

Alternatively to the previously described process, the TGF-β ligand can be presented on the EV surface, which undergoes endocytosis (Figure 1) [51]. Delivery of the TGF-β to the cell by an EV affects many aspects of its signaling and its bioactivity, including promotion of excessive TGF-β stimulation [52]. Bounded TGF-β can be carried in the lumen or on the surface of EVs in latent or active form, as evidenced by the increased phosphorylation of SMAD2 after exosome exposure. Additionally, exosome-bound TGF-β1 is retained in endosomal compartments, resulting in prolonged cell signaling compared to free TGF-β1 [53].

## 4. EV-Mediated TGF-β Signaling Pathway in the TME

**Figure 2:**
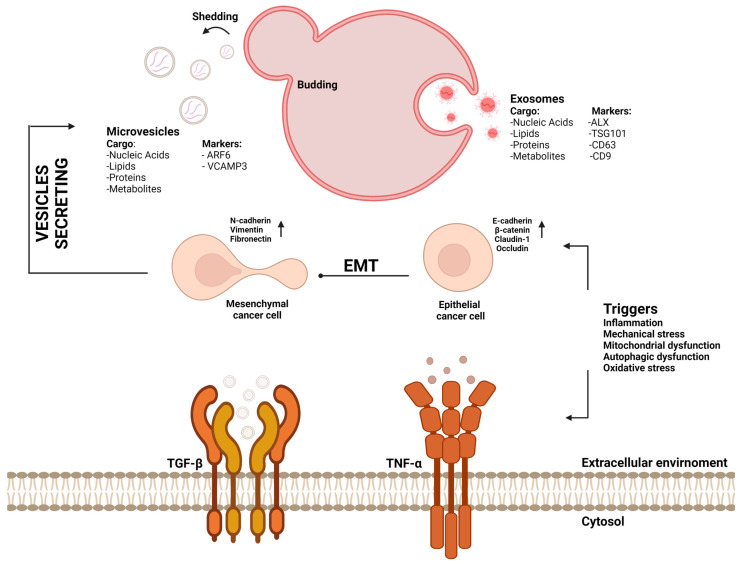
**The mechanism of EV secretion induced by TGF-β activation.** This figure describes the impact of extracellular vesicles relative to TGF-β signaling by focusing on mechanisms by which EV cargo can influence cancer formation, metastasis progression, immune evasion, and response to anticancer treatment by EMT mechanisms. The duality of interactions between TGF-β and TNF-α and the expression of these inflammatory factors induce the EMT mechanism, which is associated with EV secretion. These membrane-based populations are important communicators between tumor cells and their microenvironment. Various pathological conditions can favor the activity of TNF-α and TGF-β, among which we can distinguish mainly inflammation, mechanical stress, mitochondrial dysfunction, and oxidative stress. Exosomes or microbubbles are only selected as follicle populations in response to the described factors. During the induction of inflammation associated with increased cell migration and change in their shape, migrasomes, a population of extracellular vesicles detached from the retraction fibers of the cell during the migration process and then absorbed by neighboring cells or circulating in the extracellular matrix, may be secreted. Abbreviations: EVs, extracellular vesicles; TGF-β, transforming growth factor β; EMT, epithelial–mesenchymal transition; TNF-α, tumor necrosis factor α.

The mechanisms of TGF-β signaling are characterized by many pitfalls, preventing clear classification of its role as a promoter of carcinogenic progression or suppression. On the one hand, TGF-β inhibits early-stage hyperplasia, and on the other hand, it helps aggressive tumors to metastasize. TGF-β induces the secretion of extracellular vesicles involved in intercellular communication. Their content, based on proteins and miRNAs, regulates the expression of genes associated with neoplastic cell proliferation. EVs have been assumed as essential mediators between cancer cells within the TME. EVs can mediate and maintain molecular gradients that lead to differential responses of the various cell types that populate the TME [54,55,56]. Cancer cells respond to signals in their microenvironment and adapt to changes by going into a highly plastic state in which they are prone to be transformed into a different type of cell [57]. The described process is known as epithelial–mesenchymal transformation (EMT), which is characterized by a loss of apicobasal polarity and cell–cell adhesion the characteristic features of motile mesenchymal cells. Owing to their participation in tumorigenesis, EVs are extremely important, as they are characterized by the expression of proteins responsible for the progression of EMT [58,59]. EMT is a complex, stepwise phenomenon that favors decreasing cytokeratin and epithelial cadherin (E-cadherin) and increasing CDH2, vimentin, fibronectin, and β-catenin expression [60]. EV-specific overexpressing proteins promote EMT progression, which suggests that the EV population participates in intercellular communication, enhancing neoplastic transformation. Many studies have confirmed the important role of vimentin in the extracellular environment, indicating its participation in EVs. Owing to location where its expression intensifies, it has even been referred to as extracellular vimentin. Packing vimentin inside EVs is supported by the activity of GTPase from the Ras protein family, which is a proto-oncogene. Increased EMT characterizes cells that are targeted by EVs containing this protein in their interior upon cargo adsorption. TGF-β is the factor stimulating the increase in the extracellular expression of vimentin. Thus, the inflammatory response signaling pathway induced by TGF-β induces both EV secretion and enhances vimentin expression, contributing to the participation of EVs in tumor progression (Figure 2) [61,62,63].

## 5. The Relevance of the TGF-β Paradox in the EMT Mechanism

TGF-β carried by EVs is a known factor inducing the initiation and progression of the EMT process. The mysterious dual nature of TGF-β is mainly observed when the tumor growth suppressor acquires the features of an oncogenic cytokine and promotes invasion and metastasis [64,65]. TGF-β is the most potent inducer of the Snail transcription factor, which is responsible for upregulating the expression of proinflammatory mediators, i.e., interleukin-1, -6, and -8 (IL-1, IL-6, and IL-8, respectively) [66]. It is associated with a decrease in E-cadherin expression and an increase in vimentin, indicating a direct interaction between EMT and tumor progression. TGF-β affects blood vessel cells, including vascular ECs, inducing a downstream signaling pathway mediated by type I and type II serine/threonine kinase receptors and intracellular SMAD signal transducer proteins [67]. The ECs in the newly formed tumor vascular loops are abnormal in shape and size, have wide intercellular junctions, are irregular, and have a leaky basement membrane. Moreover, such vessels are characterized by leakage of plasminogen, fibrinogen, and platelets, leading to extracellular fibrin deposition and coagulation (Figure 2) [68].

In contrast to the well-organized physiological vessels, tumor vessels show incomplete arteriovenous differentiation and incomplete differentiation of the perivascular space. Blood flow varies temporally and spatially. There is also a lack of pericytes in the tumor vessels, which inhibit further capillary formation under physiological conditions [69,70,71], which is important, owing to the participation of ECs in active angiogenesis, which, together with EMT, is the main factor leading to tumor progression. The characteristic expression of TGF-β on the EV surface can stimulate or inhibit angiogenesis via differential surface receptors directly associated with carcinogenesis [72]. The content of EVs generates the neoplastic cell phenotype based on the transfer of functional molecules or oncoproteins and the activation of other signaling pathways promoting carcinogenesis. The binding of EVs with cells that absorb their contents inhibits the response of immune cells, allowing for the generation of a phenotype conductive to the progression and spread of cancer cells [73]. Additionally, these protumorigenic properties are promoted by the TGF-β1-mediated differentiation of resident fibroblasts into cancer-associated fibroblasts (CAFs). It is now well established that CAFs are the most abundant cells in the TME and strongly influence surrounding cells and support tumor growth by secreting proinflammatory cytokines, chemokines, and a set of growth factors [74,75]. Fibroblast activation can occur through both the EMT and the EndMT process, in which TGF-β is involved, as mentioned earlier. Hence, blood vessel cells and epithelial cells can also obtain the myofibroblastic phenotype, become CAFs, and initiate the crosstalk with tumor cells, which may be further regulated by EV secretion. Accordingly, cancer-derived exosomes enhance the transdifferentiation of CAFs via TGF-β1, Gm26809, miRNA, long non-coding RNA (lncRNA) transport and activation of TGF-β, nuclear factor kappa-light-chain-enhancer of activated B cells (NF-κB), signal transducers and activators of transcription 3 (STAT3), and mitogen-activated protein kinase (MAPK) signaling cascades [76,77].

However, in the tumor suppression mechanism, a decrease in the activity of the RAS oncogen family of small GTPase members (RAB27a) was observed, which is directly related to the disturbance of EV biogenesis [78]. Apart from TGF-β, EVs cargo can also consist of TNF-α, IL-6, and matrix metalloproteinases (MMPs), increasing the proliferation, migration, and growth of cancer cells [3]. In addition, EVs transport fibronectin, which, upon binding to integrin receptors of CAFs, promotes tumor cell growth, enhances angiogenesis, and contributes to ECM remodeling [79]. Acting in tandem with an integrin receptor, fibronectin initiates a cascade of events that leads to the transmission of signals from the external environment to the interior of the cell, regulating the organization of the cytoskeleton [80].

## 6. The Importance of Signaling Molecules Constituting EV Cargo in the TGF-β Signaling Pathway

TGF-β overexpression induces miR-191 expression and stimulates its loading into EVs, promoting EMT progression in mesenchymal lung adenocarcinoma cells. In the case of pulmonary adenocarcinoma, the environment of precancerous bronchial epithelial cells secreting intercellular communicators in the form of EVs manifesting the presence of miR-19b and miR-92a also impacts tumor proliferation and neoplasm growth, owing to the reduction in TGF-βRI and TGF-βRII expression on the surface of target cells. In the case of colorectal cancer, it was found that TGF-β1 increased the levels of miR200b by acting on the miRNA339-untranslated region (UTR) of p27 mRNA, which inhibited the expression of cell cycle inhibitors and consequently led to increased proliferation of cancer cells [80,81]. The protumor properties associated with the TGF-βRI receptor are also observed in oral cancer neoplastic cells, where it induces the presence of miR-142-3p inside EVs, leading to increased proliferation of neoplastic cells. TGF-β signaling that promotes EV secretion is directly linked to the regulation of many of the transcription factors, including Snail family transcriptional repressors 1 and 2 (SNAI1 and SNAI2), zinc finger E-Box binding homeoboxes 1 and 2(ZEB1, ZEB2), and Twist family BHLH transcription factors 1 and 2 (TWIST1 and TWIST2). The activity of these factors is highly conducive to EMT progression [82]. In the lung cancer case, exposure to the TGF-β receptor was associated with an invasive change in EV charge, enriching it with miR-23a. The presence of miR-23a is associated with a change in the protein profile of cancer cells characterized by an increase in β-catenin levels and decreased expression of E-cadherin [83,84]. In the case of pancreatic ductal adenocarcinoma cells, TGF-β-dependent secretion of EVs implies the expression of highly upregulated in liver cancer (HULC) long non-coding RNAs (lncRNA), which strongly supports the transformation of cells into the mesenchymal phenotype. The impressive properties of EVs have allowed them to be used in experimental research based on the assessment of neoplastic induction by EV cargo. In particular, EVs secreted by stem cells are characterized by the ability to transform normal and precancerous epithelial cells with the help of their charge. For example, EVs released by mesenchymal stem cells triggered phenotypic changes in epithelial cells. Their action is based on the direct inhibition of androgen receptor signaling and activation of the TGF-β signaling pathway [51]. EVs secreted by stem cells are characterized by the expression of miRNAs miR-21, miR-31, and miR-145. EVs transmit the genetic information contained within the cells, which is associated with the generation of aggressive subpopulations.

The empirical nature of many studies allows for the manipulation of experimental conditions to evaluate the effect of EV content on the cellular environment [85,86]. The EVs that are derived by some groups of stem cells possess characteristic lncRNA, as well as metastasis-related lung adenocarcinoma transcript 1 (MALAT1), long intergenic non-protein-coding RNA (linc-ROR), transcription factor EMT SNAI2, and SRY-box transcription factor 2 (SOX2). The placement of such EVs among unchanged cells has increased their proliferative and invasive capacity. However, the data in the literature indicate one interesting phenomenon of TGF-β. EVs containing high levels of TGF-β can be secreted not only by neoplastic cells but also through normal cells [87]. These include EVs secreted in milk by the breast epithelial cells of lactating healthy women and EVs secreted by human umbilical cord mesenchymal stem cells (MSCs). Analysis of the alteration of EVs numbers and content profiles indicated that activation of canonical and non-canonical TGF-β signaling pathways triggers the ability of EVs to induce EMT in both normal and neoplastic cells. Placing cells in the presence of TGF-β-induced EVs changed their protein profile, which is characterized by the induction of phosphorylation of proteins involved in EMT (transglutaminase 2 (TGM2), annexin-A1, VCAM-1, and Chrombox3), the formation of intercellular junctions (E-cadherin and N-cadherin), and the interaction of cells with the ECM through matrix metallopeptidases 2 and 9 (MMP-2 and MMP-9). As mentioned, tumor progression is strongly associated with the development of inflammation-induced increased migration of neoplastic cells. EVs are secreted at the leading edge of migrating cells, which affects the migration pathways of neighboring cells via specific membrane receptors. The directed migration mechanism of cancer cells can actively lead to EVs endocytosis, which drives the transmission of behaviors that promote neoplastic transformation on the other cells [88,89,90].

## 7. Conclusions

Prolonged inflammation is a key factor in tumor progression and metastasis. This process may be modulated by EVs secreted by cancer cells, highlighting the importance of vesicle cargo in the course of carcinogenesis. The explanation for this phenomenon is the activation of inflammatory signaling pathways, which not only affects the type of released EVs but can also affect their quantity and content. EVs are a mixture of heterogeneous structures classified according to their size and the method of their biogenesis. Accurate identification of a specific type of EV is difficult, owing to the lack of unambiguous markers characterizing a specific group of membrane structures. EV-transported cargo can alter the functions of cytokines and their downstream molecular effectors, as exemplified by TGF-β signaling. Accordingly, cancer cells induce a proinflammatory phenotype of normal cells, such as ECs, affecting their adhesive and migratory properties. In this review, we identified specific types of EVs containing in their cargo miRNA particles and receptors specific to the TGF-β pathway, representing a significant contribution to the development of research aimed at understanding the mechanisms of cellular signaling regulation, which is important for tumorigenesis, as well as other chronic inflammatory disorders.

## Data Availability

Not applicable.
